# Myocardial Ischemia and Diabetes Mellitus: Role of Oxidative Stress in the Connection between Cardiac Metabolism and Coronary Blood Flow

**DOI:** 10.1155/2019/9489826

**Published:** 2019-04-04

**Authors:** Paolo Severino, Andrea D'Amato, Lucrezia Netti, Mariateresa Pucci, Fabio Infusino, Viviana Maestrini, Massimo Mancone, Francesco Fedele

**Affiliations:** Department of Cardiovascular, Respiratory, Nephrology, Anesthesiology and Geriatric Sciences, Sapienza University of Rome, 00161 Rome, Italy

## Abstract

Ischemic heart disease (IHD) has several risk factors, among which diabetes mellitus represents one of the most important. In diabetic patients, the pathophysiology of myocardial ischemia remains unclear yet: some have atherosclerotic plaque which obstructs coronary blood flow, others show myocardial ischemia due to coronary microvascular dysfunction in the absence of plaques in epicardial vessels. In the cross-talk between myocardial metabolism and coronary blood flow (CBF), ion channels have a main role, and, in diabetic patients, they are involved in the pathophysiology of IHD. The exposition to the different cardiovascular risk factors and the ischemic condition determine an imbalance of the redox state, defined as oxidative stress, which shows itself with oxidant accumulation and antioxidant deficiency. In particular, several products of myocardial metabolism, belonging to oxidative stress, may influence ion channel function, altering their capacity to modulate CBF, in response to myocardial metabolism, and predisposing to myocardial ischemia. For this reason, considering the role of oxidative and ion channels in the pathophysiology of myocardial ischemia, it is allowed to consider new therapeutic perspectives in the treatment of IHD.

## 1. Introduction

Myocardial ischemia represents a condition of sufferance for cardiomyocytes due to coronary blood flow reduction as compared to their metabolic requests, and it may exhibit through several clinical conditions [1]. From the epidemiological point of view, the mortality rate for ischemic heart disease (IHD) is about 12% of total death causes, and in a population aged between 35 and 74 years, myocardial infarction represents the main cause of death and morbidity [2]. Recent studies demonstrated that, in western countries, the mortality rate for IHD reduced over the past four decades, although it now represents one of the main causes of death in people over 35. Instead, in developing countries, the IHD death rate is expected to increase because of environmental pollution, increasing life expectancy and assumption of western habits such as western diet, smoking, alcohol assumption, and physical inactivity [3-6]. From the pathophysiological point of view, IHD may represent the consequence of both coronary artery disease (CAD) and coronary microvascular dysfunction (CMD) [7-11]. There are many regulatory mechanisms which, acting at coronary vasculature, are responsible for the adaptation of coronary blood flow (CBF) to the myocardial metabolic demand [7–10]. Ion channels represent the end effector of all these mechanisms because they regulate vassal tone through ion influx and efflux in both endothelial and smooth muscle cells [8–10]. Diabetes mellitus, such as other cardiovascular risk factors, may impair the function of these channels predisposing to CMD, and CAD and oxidative stress seem the main mechanisms through which diabetes mellitus acts [8].

## 2. Diabetes Mellitus and Oxidative Stress: Connection with Ischemic Heart Disease

### 2.1. Pathophysiological Basis of IHD

**Figure 1:**
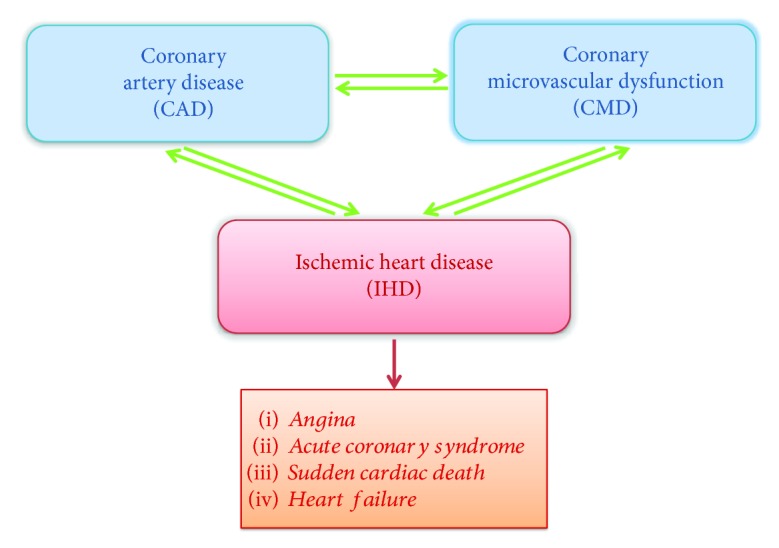
Pathophysiological basis of IHD and its clinical manifestations.

IHD may be the result of two pathophysiological mechanisms of action: CAD and CMD. CAD represents a condition defined by the presence of an atherosclerotic plaque which reduces the vessel diameter more than 50%, and it is usually the main, but not the only cause of IHD. Indeed, often the presence of CAD is not associated with the onset of IHD and conversely IHD may develop in the absence of angiographic relevant atherosclerotic plaques [7–9]. About that, the role of microcirculation may be crucial in the pathophysiology of IHD [7-11]. CMD, causing a reduced endothelial and nonendothelial response of coronary microvasculature to myocardial demands, is associated with coronary blood flow reduction and myocardial ischemia independently from CAD [10, 11]. From the opposite point of view, CMD promotes the development of atherosclerotic plaques too, altering physical coronary blood flow features and increasing epicardial vessel shear stress [7–11]. From the clinical point of view, IHD may exhibit with several conditions such as angina, acute coronary syndrome, sudden cardiac death, and heart failure [7, 9, 12–26] (Figure 1).

### 2.2. Diabetes Mellitus as Risk Factor for Ischemic Heart Disease

**Figure 2:**
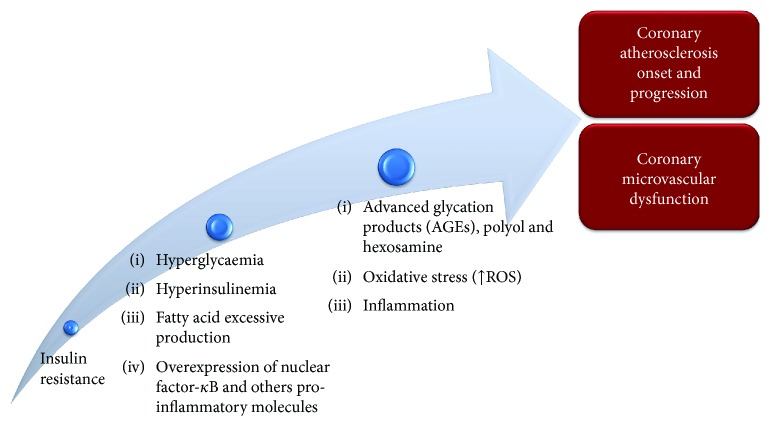
Pathophysiology of diabetes mellitus and its role in the determinism of IHD.

There are several cardiovascular risk factors which are involved in IHD and other cardiovascular diseases pathogenesis, and diabetes mellitus represents one of the most significative ones [8, 27]. Cardiovascular diseases, in particular IHD, represent the main long-term complication and death cause among diabetic patients [8]. Moreover, the risk to develop cardiovascular disease is similar for both type 2 diabetes mellitus (T2DM) and type 1 diabetes mellitus (T1DM) patients, even if there are gender and age differences between the two types [8, 27]. The main mechanism of diabetes mellitus pathophysiology is a condition of long-time insulin resistance which is strictly associated with hyperglycaemia followed by a compensatory hyperinsulinemia [27]. Hyperglycaemia, insulin resistance, and fatty acid excessive production lead to an increase in systemic oxidative stress, inflammatory response, and advanced glycation product (AGE) production [8, 27]. All these mechanisms contribute both to coronary atherosclerosis onset and progression and to coronary microvascular dysfunction [8, 27]. In particular, hyperglycaemia stimulates AGE production, accumulation of free radicals, polyol and hexosamine flux in endothelial cells, and an increase in intravascular inflammatory response through the overexpression of several factors, such as nuclear factor-*κ*B, which is initially produced by endothelial cells, and it promotes the transcription of inflammatory response-associated genes and leukocyte recruitment near the vascular wall [28]. These mechanisms are shared between diabetes mellitus and other cardiovascular risk factors, promoting dysfunction and apoptosis of endothelial cells [29, 30]. Moreover, diabetes-related renal dysfunction promotes mineral metabolism imbalance, and it determines the accumulation of calcium in coronary arteries leading to an increase in arterial rigidity and atherosclerotic plaque burden [29, 30]. Regarding myocardial ischemia in patients with diabetes mellitus, its pathophysiology is not completely understood yet. Some diabetic patients show IHD due to the presence of coronary atherosclerotic plaques which obstruct the blood flow directly to the myocardium while others develop IHD due to CMD in the absence of atherosclerotic plaques in coronary epicardial vessels [7, 9]. As regard the CMD in diabetes mellitus, oxidative stress together with hyperglycaemia and inflammation response determines coronary vasomotion alteration through the impairment of both endothelium-dependent vasodilation, reducing NO production and increasing endothelin-1 release, and endothelium-independent vasodilation [31]. Moreover, Yokoyama et al. underlined an inverse relationship among myocardial flow reserve and haemoglobin A1C average levels and fasting glucose plasma values. Authors demonstrated the role of diabetes mellitus in the determinism of CMD and myocardial ischemia [32]. Endothelial, smooth muscle cells and cardiomyocyte death, autonomic dysregulation, lipotoxicity, and endomyocardial fibrosis are other mechanisms through which diabetes mellitus promotes IHD [33, 34]. In most cases, the impact of diabetes mellitus on IHD determinism is improved by the presence of other cardiovascular risk factors such as dyslipidemia, arterial hypertension, and inflammation [35–45] (Figure 2).

### 2.3. Role of Oxidative Stress in the Pathophysiological Continuum among Diabetes Mellitus and IHD

**Figure 3:**
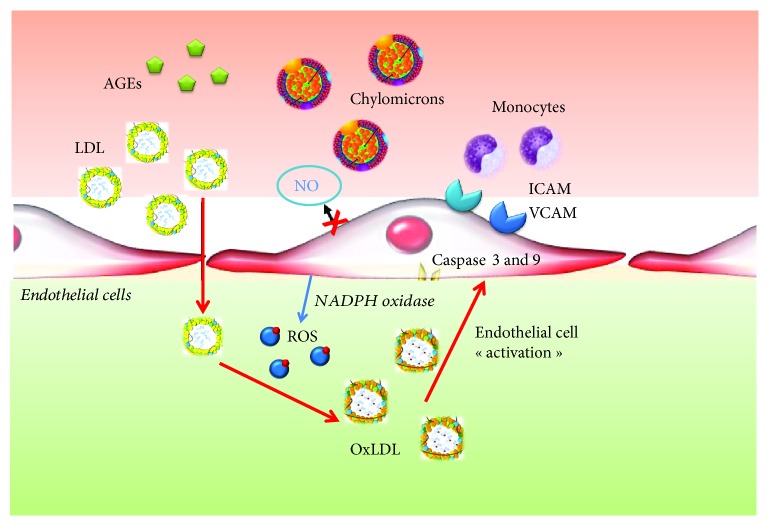
Role of oxidative stress in the pathogenesis of coronary artery disease and coronary microvascular dysfunction.

Oxidative stress is defined as a condition of oxidant molecule cellular excess compared to antioxidant ones [46]. The presence of oxidants is normally neutralized by the presence of antioxidant cell systems which include both enzymatic molecules, such as superoxide dismutase (SOD) and catalase, and nonenzymatic molecules, such as all trans-retinol 2 and ascorbic acid [46]. The activity of these systems and the regulation of redox cell state are crucial for cell function and survival. When produced in not excessive quantity, ROS are involved in several physiological mechanisms regarding the cardiovascular system [46, 47]. They stimulate angiogenesis via the vascular endothelial growth factor (VEGF) pathway, and they are involved in endothelial cell regeneration, proliferation, and migration. H2O2 is crucial for postischemic neovascularization, and they are also involved in the regulation of coronary endothelial-dependent and independent vasodilatation [7–9, 46, 47]. In pathological conditions, the damage and/or the overload of antioxidant systems make them unable to contrast the production of oxidants. Reactive oxygen species (ROS) play a central role as mediators of oxidative stress and its complications [46–48]. This term defines several agents among which there are both oxygen radicals as hydroxyl (OH-), superoxide (o2--), and peroxile (Ro2-) and several nonradical oxygen species as hydrogen peroxide (H2O2) [47, 48]. However, ROS are highly reactive molecules and in case of their accumulation, they may cause several modifications in the structure and function of DNA, proteins, and lipids [46, 48–50]. Metabolic cell activity and environmental factors, such as wrong diet and smoke, contribute to ROS production and therefore oxidative stress which may predispose to several pathological conditions as neurological disease, cancer, atherosclerosis, hypertension, diabetes mellitus, and cardiovascular diseases [46, 47]. There is an important link between oxidative stress and the development of diabetes mellitus and its complications [51–53]. Indeed, in diabetic patients there is not only an excess of ROS production but also a damage of antioxidant mechanism function and a stronger and prolonged inflammatory response [51]. In diabetes mellitus, ROS together with inflammatory response and hyperglycaemia plays a central role in the initiation and progression of vascular damage, supporting the atherosclerosis process and microvascular dysfunction [51, 54]. These mechanisms are the basis of chronic kidney injury, myocardial ischemia, and retinopathy, the most important diabetes mellitus complications [51, 54–58]. Moreover, patients with diabetes mellitus who were already treated with percutaneous coronary intervention show an increased risk to develop stent restenosis, mostly by using bare-metal stents and previous generations of drug-eluting stents [59, 60]. In diabetes mellitus, hyperglycaemia represents a stimulus for ROS production [51, 61]. Hyperglycaemia and the excess of intravascular ROS may cause not only the elevation of low density lipoproteins (LDL), chylomicrons, and total cholesterol values but also the oxidation and glycation of lipoproteins increasing their atherogenicity and accelerating the atherosclerotic process [51, 61–63]. Hyperglycaemia increases advanced glycation product (AGE) production, and Giardino et al. demonstrated that also ROS represents a stimulus for AGE synthesis [51, 61]. From another point of view, Sima et al. showed a bidirectional link between the AGE-LDL complex and ROS, demonstrating that the AGE- LDL complex may stimulate ROS production and the subsequent inflammatory response activation which contributes to vascular damage, through the expression of IL-1*β* and TNF-*α* [51, 62]. There is a clear link among ROS, AGE, and oxidized LDL (Ox-LDL) [51, 64, 65]. Indeed, beyond the bidirectional link between AGE and ROS, these two agents stimulate the oxidation of LDL. Ox-LDL causes a reduction in endothelial nitric oxide production and through the activation of caspase-3 and 9 stimulates endothelial cell apoptosis [51, 66, 67]. ROS contribute to atherosclerosis, also inducing the worsening of endothelial dysfunction, increasing the expression of adhesion molecules like intercellular adhesion molecule-1 (ICAM-1) and vascular adhesion molecule-1 (VCAM-1), and modulating the expression of different growth factors important in the proliferation of vascular smooth muscle cells (VSMCs) [68]. In diabetic patients, the main source of intravascular ROS is NADPH oxidase (NOS) whose expression is highly increased compared to nondiabetic patients [51, 69]. There are 4 isoforms of NOX, NOX1, NOX2, NOX4, and NOX5, which are overexpressed and play a crucial role in atherosclerosis progression in diabetic patients. NOX1 is expressed by endothelial cells. A decreased expression of NOX1 is associated with reduced leucocyte vascular wall adhesion and macrophage recruitment [51, 70]. NOX4 is expressed by endothelial and muscle cells, and it has a protective role for the wall vessel. The reduction in its expression supports the increase in inflammatory marker production such as IL-1 and MCP-1 and the progression of atherosclerosis [51, 71]. Moreover, the reduced expression of NOX4 on smooth muscle cells associates with reduced contractile gene expression and higher production and deposition of collagen [51, 72]. NOX5 may alter endothelial nitric oxide synthase (eNOS) activity contributing to endothelial dysfunction [51, 73, 74]. Inside the cell, the most important site of free radical production is mitochondria because they represent the energetic central point of the cell. Glucose from the blood circle enters inside the cell to be used for adenosine triphosphate (ATP) production. During glycolysis, pyruvate, ATP, nicotinamide adenine dinucleotide (NADH), and flavin adenine dinucleotide (FADH2) are produced. NADH and FADH2 are transferred inside mitochondria, and they have a role as electronic donors during oxidative phosphorylation. In the hyperglycaemic state, a lot of electrons are lost in the mitochondrial respiratory chain which become the most important source of the overproduction of O2- [75, 76]. Moreover, Azumi et al. highlighted an association between ROS production in human atherosclerotic coronary arteries and the NAPDH-oxidase subunit p22 phox [77]. Hyperglycaemia increases diacylglycerol (DAG) content by the activation of phospholipase C or D, which activates protein kinase C (PKC) [77]. PKC activates NADPH oxidase. The NADPH oxidase complex consists of the cytosolic components p47phox, p67phox, p40phox; a low-molecular-weight G-protein, Rac 1 or Rac 2; and the membrane-associated NOX2 and p22phox [77]. Activation of the enzyme complex requires translocation of the cytosolic components to the plasma membrane, and their association to NOX2 produces ROS [77]. Recently, particular attention was focused on the role of microRNA as a mediator of oxidative stress effects in the pathophysiology of diabetes mellitus and its complications [78–89] (Figure 3).

MicroRNAs (miRNAs) may play a role also in the regulation of protein expression such as ion channels and their subunits [78–89]. miRNAs represent noncoding RNA molecules of 21-23 nucleotides, and they are negative regulators of gene expression, modulating the stability of several messenger RNAs (mRNAs) before their translation in amino acids. Given that, miRNA takes part in several biological mechanisms such as apoptosis, proliferation, and differentiation, and it is clear how they may be also involved in pathological processes [78]. Several stimuli such as H2O2, ultraviolet (UV), and ionizing radiation may induce both ROS production and modification in miRNA expression [78, 79]. Magenta et al. demonstrated the strong upregulation of the miR-200 family in endothelial cells exposed to oxidative stress induced by hyperglycaemia and hyperlipidemia [78]. However, the miR-200 family may act also through another pathway. They reduce the p38alpha mitogen-activated protein (MAP) kinase expression, a protein involved in the regulation of the cellular cycle also important as an oxidative stress sensor [78, 80, 81]. Silent mating type information regulation 2 homolog (SIRT1) represents a histone deacetylase which is able to induce a lot of stress-responsive transcription factors, and it has also a strong anti-inflammatory and antioxidative effect for endothelial cells [78, 82]. In atherosclerosis, miR-217 and miR-200 targets SIRT1 causing its downregulation in endothelial cells. The reduction in SIRT1 expression is associated with senescence, apoptosis, and therefore endothelial dysfunction [78, 82]. miR-21 is upregulated in vascular smooth muscle cells in conditions of shear stress, and it protects cells from death through binding with programmed cell death 4 (PDCD4) [78, 82]. It determines an increase in NO production via activation of eNOS, but at the same time it reduces the expression of SOD-2 [78]. In case of ischemia, the reduced oxygen tension inside the cells determines the hypoxia-inducible factor (HIF) which is a transcription factor family involved in the shift from aerobic to anaerobic metabolism [78, 82–89].

## 3. Oxidative Stress and Ion Channel Function in the Regulation of Coronary Blood Flow

### 3.1. Coronary Blood Flow and Its Regulation

**Figure 4:**
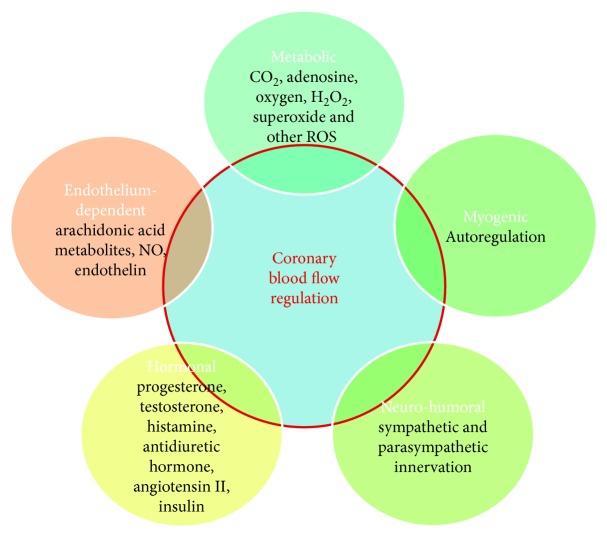
Different mechanisms involved in coronary blood flow regulation.

CBF has to satisfy myocardial metabolic and oxygen requests which continuously vary through a fine modulation of coronary resistances [90]. Microcirculation, characterized by small arteries and arterioles with a diameter included between 50 and 200 *μ*m, represents the most important site of coronary total resistance regulation [9]. In the “cross-talk” between myocardium and coronary artery circulation, several mechanisms of vasal tone regulation act to guarantee an adequate CBF to the myocardium [7–9, 90] and their contribution changes according to the considered district [7–9, 90]. Microcirculation, which represents the distal district of coronary arterial circulation, is the main site where metabolic and myogenic regulation mechanisms act, while the epicardial artery district, which represents the proximal district of coronary arterial circulation, is the main site where neurohumoral and shear stress-related regulation mechanisms act [9]. At rest, the myocardium extracts about 80% from coronary circulation and the oxygen consumption amount to 10 mL of oxygen, per minute, per gram of myocardial tissue [90]. When myocardial oxygen consumption increases, coronary circulation has to modulate its vasal tone to guarantee an adequate CBF to the myocardium. For these reasons, several vasal tone regulation mechanisms exist. Neurohumoral regulation acts through sympathetic and parasympathetic innervation which are both expressed on coronary arteries, and through their tonic activity, they determine vascular basal tone at rest [8, 9, 90]. The endothelium participates for CBF regulation producing several molecules with paracrine effects such as arachidonic acid metabolites and NO, which contributes to vasodilatation and endothelin which contribute to vasoconstriction [8, 9, 90]. Autoregulation acts myogenically, which, reducing vasal wall stress, guarantees a constant and sufficient CBF to the myocardium [8, 9, 90]. Myogenic response is mediated by variation of calcium values in smooth muscle cells which modulate their state of contraction [8, 9, 90]. CBF is also modulated by several hormones such as progesterone, testosterone, histamine, and antidiuretic hormone (ADH) which are vasodilators and angiotensin II which is a vasoconstrictor [91–94]. Insulin mediates both vasoconstriction, via activation of sympathetic fibers, and vasodilatation, via NO production stimulation [91]. Metabolic regulation acts mainly at microcirculation, and it is important for the quick adaptation of CBF to myocardial metabolic demand. The effect of metabolic regulation is mediated by several molecules produced by cardiomyocytes whose targets are represented by specific receptors and ion channels. Among these molecules, there are carbon dioxide (CO2), adenosine, oxygen, H2O2, superoxide, and other reactive oxygen species [90, 95–97] (Figure 4).

### 3.2. Coronary Ion Channels and Their Physiological Role

**Figure 5:**
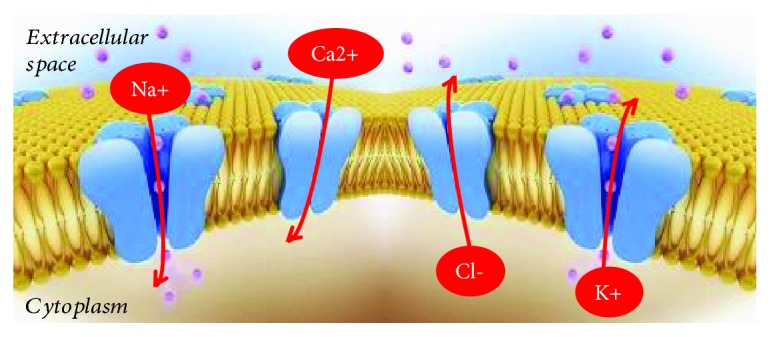
Schematic representation of ion movements through coronary ion channels.

Coronary ion channels represent the crucial connectors in the cross-talk between myocardial metabolic demand and coronary blood flow regulation. They are the final effectors of several CBF regulatory mechanisms (nervous, metabolic, endothelial, and myogenic) which act in response to myocardial metabolism variations [8, 9, 90]. In coronary circulation, ion channels are expressed both by endothelial cells where they modulate the secretion of different vasoactive substances, among which there is nitric oxide (NO), and by arterial smooth muscle cells where they regulate the vascular tone, modulating ions fluxes through the cell membrane. The importance of ion channels in the regulation of coronary blood flow and the connection between their function and IHD was also underlined by us [7–9] with particular attention for several specific single-nucleotide polymorphisms (SNPs) of genes encoding for ion channel constitutive proteins. There are several types of ion channels involved in the regulation of vasal tone and endothelial function. Voltage-gated sodium channels are associated with a late Na^+^ current which determines cell depolarization. The main function of these channels is to modulate endothelial NO production and release via endothelial Ca^2+^ levels and Na^+^/Ca^2+^ exchange regulation [8, 98]. Vascular smooth muscle cells express chloride channels, and they can be both Ca^2+^- and voltage-dependent. When these channels are opened, a Cl^−^ current moves out from the cells determining their depolarization and therefore vasoconstriction [8, 98]. Chloride channels determine the opposite effect when they are closed [9]. One of the main channels involved in the regulation of microvascular resistance are voltage-gated calcium channels (Cav). They regulate the Ca^2+^ current from the extracellular to intracellular environment. Their final effect is to increase vascular tone and determine a reduction of CBF [9]. Potassium channels are expressed both by endothelial cells, where they modulate the secretion of vasoactive substances such as NO, and by arterial smooth muscle cells, where they regulate cell state of contraction [7, 8]. The opening of the potassium channel determines the efflux of K^+^ from the intracellular to extracellular environment, the membrane resting potential moves to more negative values, the cell is hyperpolarized, and the Ca^2+^ channels are closed. The final effect of this event is artery vasodilatation, thanks to smooth muscle cell relaxation. The closing of potassium channels, instead, determines cell depolarization and the activation of voltage-gated Ca^2+^ channels which determines the increase in calcium cell concentration. The final effect of this event is smooth muscle cell contraction and the increase in vasal tone [90]. In the coronary circulation, four types of potassium channels are described in literature: KATP, KCa, Kv, and inward rectifier potassium (Kir) channels. KATP channels are made up of two subunits: an inward rectifier-potassium channel (Kir subunit) and an ATP-binding cassette protein defined as sulfonylurea-binding subunit (SUR) [7, 8]. Kir subunits have a crucial role in maintaining resting membrane potential because they support a faster inward K^+^ current than an outward one while SUR subunits bind ATP [7, 8]. Coronary KATP channels are involved in the metabolic regulation of coronary vascular tone [7, 8]. KATP channels open when intracellular ATP is reduced, and they allow the efflux of K^+^ from the intracellular to extracellular environment [7, 8]. This condition associates with reduction of intracellular Ca^2+^ values and therefore vasodilatation [7]. KATP channels are mainly closed in normal metabolic conditions [7, 8]. The main represented KATP subunit combinations in coronary circulation are Kir6.2/SUR2A and Kir6.1/SUR2B [8]. Kv channels regulate CBF at rest and during cardiac stimulation [7, 8]. They are the targets of several vasoactive molecules, and for this reason they are involved in endothelial-dependent and -independent vasodilatation [8, 99]. Vasodilating molecules open Kv via the cAMP-dependent pathway while vasoconstrictor ones close Kv, increasing Ca^2+^ cell levels [8, 99]. Several channels of the Kv family, such as Kv1.5 and Kv1.3, are involved in H2O2-mediated CBF regulation [95, 100]. KCa channels are expressed by both endothelial and smooth muscle cells, and they have a crucial role in preserving the rest membrane potential [7, 8]. KCa channel activation, associated with the efflux of K^+^ from the intracellular to extracellular space, is caused by two main stimuli: the increase in intracellular Ca^2+^ levels and membrane depolarization [7, 8, 90]. Three types of KCa channels are described in coronary artery circulation [7, 8, 90]. On the basis of their conductance, they are divided into small (S), intermediate (I), and big (B). KCa channels are redox-sensitive ones; in particular, they contribute to vasodilatation in response to endothelial-derived hyperpolarizing factor (EDHF), lipoxygenase metabolites, and H2O2 [101–104]. However, they are also involved in vasoconstriction, because they represent the target of several vasoconstrictor agents such as endothelin and angiotensin II which determine their inhibition [8, 105–108] (Figure 5 and Table 1).

Transient receptor potential vanilloid 1 (TRPV1) channels belong to the vanilloid TRP family, and they have permeability to several cations such as Mg^2+^, Ca^2+^, H^+^, and Na^+^ [109, 110]. In the cardiovascular system, TRPV1 channels are expressed by endothelial smooth muscle cells, by the myocardium, and by nerve myocardium nerve fibers [7, 8, 90]. TRPV1 channel activation is associated with coronary vasodilatation; they contrast atherosclerosis onset and progression and vascular and myocardium remodeling, and they reduce arterial pressure values, inducing endothelial NO release [109]. TRPV1 activation plays an important role against vascular oxidative stress effect because they increase mitochondrial Sirtuin 3, UCP2, and PPAR-*γ* expression [109].

### 3.3. Impact of Oxidative Stress on Ion Channel Function

**Figure 6:**
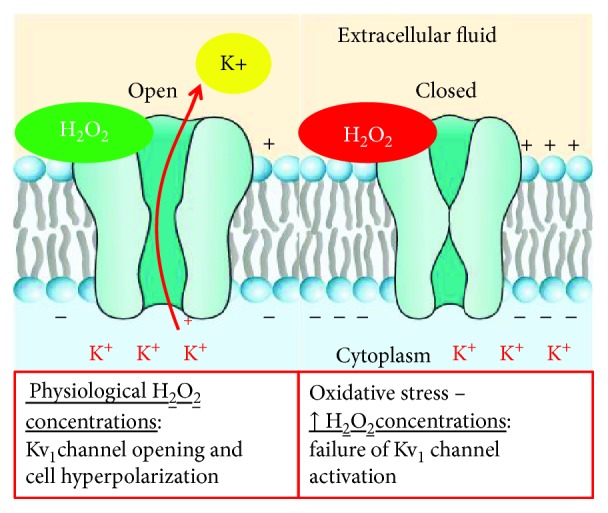
Physiological and pathophysiological roles of H2O2 on voltage-dependent potassium channel (Kv).

Some products of myocardial metabolism mediate CBF according to myocardium metabolic demand. In this cross-talk, ROS, produced in not excessive quantities, may have an important physiological role interacting with ion channel function. H2O2 is a product of myocardial metabolism which is involved in coronary autoregulation [111]. It may represent an endothelial hyperpolarizing factor [112] which causes coronary dilatation [104] through its activation of K+ channels. Saitoh et al. demonstrated that H2O2, produced in relation to cardiomyocyte oxygen consumption, represents a stimulus for arteriole dilatation and coronary blood flow increase [97]. They confirmed the role of H2O2 because they showed that catalase and 4-aminopyridine (4-AP) intracoronary infusion is associated with the intracoronary H2O2 levels and coronary blood flow reduction [97]. H2O2 may cause the modification of K^+^ currents [97]. H2O2 and probably other types of ROS may also stimulate endothelial-independent vasodilatation because they act as smooth muscle BKca channel openers through a redox-induced G protein dimerization [90]. About that, several studies suggested a role for large-conductance Ca^2+^/voltage-sensitive K^+^ channels (BKCa) as a target of H2O2 [96, 101, 102, 104]. However, the only H2O2 activity on BKCa was not enough to cause coronary vasodilatation [97]. Kv channels may represent the main ones involved in ROS-mediated coronary vasodilatation [90] Rogers et al. studied other possible targets of H2O2 that may be involved in redox-mediated coronary vasodilatation [96]. They previously demonstrated that 4-AP, a voltage-gated K^+^ channel (Kv) inhibitor, reduced H2O2 production and coronary vasodilatation [97]. For this reason, they focused on the possible role of the Kv channel as a redox-sensitive regulator of coronary blood flow [96]. Their results suggest that H2O2 acts through thiol oxidation and its effect on Kv channels developed quickly (2-3 minutes) [96] (Figure 6).

**Figure 7:**
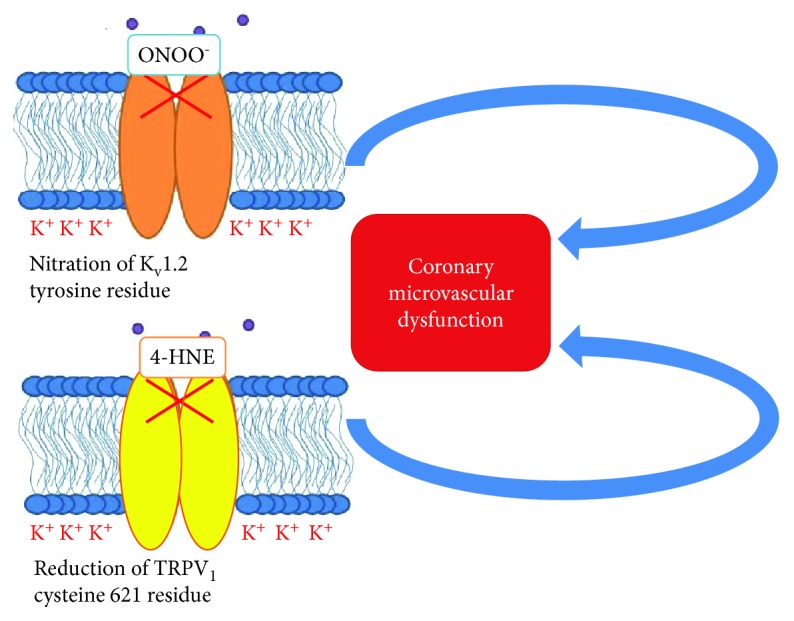
An excess of peroxynitrite (ONOO-) may lead to Kv-mediated vasodilatation impairment through Kv1.2 tyrosine residue nitration; 4-hydroxynonenal (4-HNE) targets a cysteine 621 residue of the TRPV1 coronary channel impairing its function and contributing to CMD in diabetes mellitus.

Thiol groups are probably contained in proteins involved in Kv channel regulation or they are inside the molecular structure of channels [96, 97]. Moreover, the intracoronary infusion of DTT, a thiol reductant, and NEM, a thiol-alkylating agent, reduces the effect of H2O2 on Kv channel activity [96]. As we previously described, ion channels play a crucial role in the cross-talk between myocardial metabolic demand and coronary blood flow [7–9]. The Kv channel family is expressed on endothelial and smooth muscle cells, and it fulfilled the role of coronary blood flow metabolic regulators [7–9, 90]. These channels are redox-sensitive ones, and H2O2, produced by mitochondria, determines their opening and the following cellular hyperpolarization which is associated with vascular dilatation [96, 97, 100]. Ohanyan et al. focused their attention on the Kv1.5 channels which are mainly expressed on smooth muscle cells, and they are both redox- and oxygen-sensitive channels [100]. They showed that Kv1.5 ^−/−^ mice had an impaired response of coronary blood flow to the increased myocardial work, leading to myocardial ischemia and heart pump failure through microvascular dysfunction and without the presence of atherosclerotic obstructive plaques [100]. Myocardial ischemia represents the final effect of Kv1.5 dysfunction [100]. Indeed, after the noradrenaline infusion in Kv1.5 ^−/−^ mice, there is an imbalance between oxygen delivery and oxygen consumption caused by the impaired response of coronary circulation, which is unable to sustain coronary arterial pressure and heart pump, to the increased cardiac work [100]. Myocardial ischemia, pump efficiency, and arterial pressure reduction do not occur in wild-type mice [100]. However, Kv1.5 activity is not the only mechanism which guarantees an adequate blood flow in response to myocardial work [100]. There are other ion channels and mechanisms involved in this [90, 100]. Indeed, the absence of Kv1.5 in mice facilitates myocardial ischemia, but it is not lethal, coronary dilatation in response to H2O2 is not completely abolished in Kv1.5^−/−^ mice, and moreover in Kv1.5^−/−^ mice there is an upregulation of Kir6.2, Kir6.1, and Kv1.2, suggesting a compensative role of these channels for coronary blood flow regulation in the absence of Kv1.5 [100]. For this reason, it may be other ion channels involved in the oxygen and redox-sensitive coronary blood flow regulation [100, 113, 114]. For this reason, Ohanyan et al. focused on Kv1.3 channels and they demonstrated that these channels have a crucial role in the connection between cardiac metabolism and coronary blood flow [95]. Kv1.3 channels participate in H2O2-induced vasodilatation, but they did not involve in that one induced by adenosine and acetylcholine [95]. Indeed, Kv1.3^−/−^ mice showed an impaired blood flow regulation in response to cardiac work increase [95]. Moreover, administration of correolide, a blocker of the Kv cannel family, in wild-type mice reproduces the same condition seen in Kv1.3^−/−^ mice [95]. In the study of Ohanyan et al., Kv1.5^−/−^ mice developed heart pump failure when they underwent a growing cardiac work [100]. In this case, coronary microvascular dysfunction and not the presence of an obstructive atherosclerotic plaque provoked heart failure. This study supports results [7–9] about the importance of microcirculation in the pathophysiology of IHD [115]. In obese and diabetic patients, diastolic dysfunction represents an early and frequent abnormality of heart function [116–118]. Insulin resistance and the renin angiotensin-aldosterone system (RAAS) had a crucial role in the determinism of diabetes complications and diastolic heart failure [116, 119, 120]. Jia et al. defined the role of coronary microvascular dysfunction in the determinism of diastolic heart failure in diabetes mellitus [116]. They demonstrated that administration of typical west diet in mice was associated with cardiac remodeling and fibrosis, accumulation of M1-polarized macrophages, and reduced occluding and claudin-5 expression, which belong to endothelial tight junctions, and they are markers of endothelium permeability [116]. However, their most important finding was that in these mice there was an upregulation of the endothelium epithelial sodium channel (EnNaC) [116]. In diabetic and obese female mice, the excess in dietary intake caused an overexpression of cardiac mineral-corticoid receptors which together with heightened oxidative stress and inflammation led to EnNaC overexpression, a condition which caused diastolic heart failure through microvascular dysfunction [116, 121, 122]. EnNaC determined the excessive endothelial intake of Na^+^ which caused the reduction of NO production [123] and the polymerization of G-actin to F-actin that determined arterial stiffness [124]. The administration of low doses of amiloride, an antagonist of EnNaC, reduced the risk of LV diastolic heart failure in diabetic and obese female mice [116]. Dwenger et al. studied Kv1 channels, and they confirmed the crucial role of this type of channels in the connection between myocardial metabolism and coronary blood flow [125]. Moreover, they focused on the redox sensibility of these channels [125]. The regulation of Kv1 channel functions is determined by several posttranslational modifications on cysteine, tyrosine, and methionine residues belonging to the channel structure [125, 126]. A dual role of oxidative stress on Kv1 channels was supposed [125, 126]. Indeed, physiological levels of H2O2 represented a stimulus for channel activation and K^+^ peak increase, conditions associated with smooth muscle cell hyperpolarization and coronary vascular dilatation [125, 127]. However, in condition of heightened H2O2 production such as diabetes mellitus, it failed Kv1 channel activation and it even determined their closure [125]. Peroxynitrite (ONOO^−^) is produced from superoxide and NO [128]. Li et al. demonstrated that an excess of ONOO^−^ led to Kv-mediated vasodilatation impairment through Kv1.2 tyrosine residue nitration [129]. Several authors focused on the role of the TRP channel family in the regulation of coronary blood flow in response to oxidative stress [130–132]. Guarini et al. had already demonstrated that the transient receptor potential vanilloid 1 (TRPV1) channels, belonging to the TRP channel family, are impaired in diabetic mice and they contributed to the development of microvascular dysfunction in these models [130, 133]. TRPV1 is expressed by the endothelium of coronary vasculature, and it represents an oxidative sensor which regulates coronary blood flow in relation to myocardial redox state [130, 132]. In diabetic patients, the persistent exposure to oxidative stress promotes lipid peroxidation and its by-product formation such as 4-hydroxynonenal (4-HNE) which causes posttranslational modifications through the interaction with several amino acid residues contained in the ion channel structure [130]. 4-HNE had a main role in the determinism of cardiomyocyte hypertrophy, onset and progression of atherosclerotic disease, and ischemia-reperfusion damage after myocardial ischemia [130, 134, 135]. DelloStritto et al. confirmed the contribution of 4-HNE also in the determinism of microvascular dysfunction in diabetes mellitus [130]. In particular, they demonstrated that the target of 4-HNE on the TRPV1 channel was the cysteine 621 residue. The final effect was the reduction of TRPV1-dependent coronary blood flow dilatation which may contribute to microvascular dysfunction in diabetes [130] (Figure 7).

## 4. Ion Channels as Target in the Therapy against Ischemic Heart Disease

Among potassium channels, KATP represents the main pharmacological target in the treatment of diabetes mellitus and cardiovascular diseases. In this context, it is involved in several pathophysiological processes, and for this reason, it shows a remarkable therapeutic potential [136]. In diabetic patients, pancreatic *β*-cell KATP, in particular SUR subunits, represents a target of sulphonylureas which act as antagonist of this channel, causing their closure [136]. Sulphonylureas promotes *β*-cell depolarization and the increase in insulin secretion [136]. Diazoxide is a Kir6.2/SUR1 KATP channel opener, and it is used in hypertensive crisis [136–139]. Pinacidil and cromakalim are Kir6.2/SUR2A KATP and Kir6.2/SUR2B KATP channel openers, and they determine arteriole resistance reduction, arterial blood pressure reduction, and vasodilatation [136–139]. In patients with IHD, coronary smooth muscle and myocardial KATP become therapeutic targets of several molecules such as nicorandil and levosimendan which cause the opening of these channels, a condition associated with higher CBF and better myocardial perfusion [140]. Nicorandil has a nitrate-like effect, and it also blocks calcium channels; for this reason, it is used in the treatment of stable angina [140]. Nicorandil may reduce the possibility of QT abnormalities and ventricular fibrillation in patients who underwent coronary angioplasty after acute myocardial infarction, and it is also used in the management of no-reflow phenomenon which may manifest after the same procedure [141]. Zhang et al. demonstrated that nicorandil, stimulating M2 macrophage polarization and inhibiting M1 macrophage polarization, reduces macrophage phagocytic activity and ROS and cytokine production [140]. Moreover, they demonstrated that nicorandil promotes endothelial reconstitution because, promoting M2 macrophage polarization, it increased VEGFA expression which has a proangiogenic effect [140]. In particular, nicorandil ameliorates cell redox state reducing ROS production and increasing mitochondrial membrane stability and Bcl-2/Bax ratio. NF-*κ*B which is made up of two subunits, p50 and p65, represents a nuclear factor which promotes the transcription of several genes involved in inflammatory response and in M1 macrophage polarization [140]. Kupatt et al. demonstrated that NF-*κ*B signaling pathway upregulation may aggravate myocardial ischemic damage [140, 142]. Nicorandil reduces NF-*κ*B pathway activity, acting through the inhibition of the p65 subunit and therefore M1 macrophage polarization [140]. Moreover, angina IONA study demonstrated that nicorandil improved the prognosis of patients with stable angina [143]. Levosimendan beyond the action on KATP represents a calcium sensitizer, and it determines a remarkable reduction in pulmonary capillary wedge pressure in a patient who presents with heart failure with low output [136, 144]. Moreover, levosimendan has electrophysiological effects such as inhibitors of phosphodiesterase [145]. It is used to improve heart pump function, and it reduces the risk to develop arrhythmic events more than milrinone, after myocardial ischemia [146]. Bunte al. demonstrated that preconditioning with levosimendan may reduce myocardial ischemic area by about 50% [145]. This effect may be due to the activation of mBKCa channels by levosimendan. mBKCa channels are voltage-gated potassium channels involved in the regulation of intracellular calcium homeostasis and which are expressed on the inner mitochondrial membrane [145, 147, 148]. In levosimendan preconditioning, coronary KATP channels seem to play an important role because their activation is associated with vasodilatation and increase in CBF [145]. Intermediate and small-conductance calcium-activated K^+^ channels (IKCa and SKCa) have an important role in the metabolic regulation of coronary vasal tone, and they mediate endothelial NO release [149]. The impairment of the function of these channels by diabetes mellitus and other cardiovascular risk factors may contribute to endothelial dysfunction [149]. Wang et al. demonstrated that ischemia-reperfusion damage inhibits the IKCa and SKCa function impairing coronary EDHF-mediated vasodilatation, and it reduced TRPC3 expression, a channel belonging to the TRP family and which is involved in the metabolic regulation of CBF, through a calcium-related pathway, both stimulating NO release and mediating IKCa and SKCa function [150]. Wang et al. focused on the close relation between IKCa and SKCa channel dysfunction and TRPC3 channel impairment in the determinism of endothelial dysfunction and coronary microvascular one [150]. For this reason, they proposed TRPC3 as a new therapeutic target to improve CBF in ischemic conditions [150]. Isosteviol is a natural sweetener contained in Stevia rebaudiana Bertoni leaves, and from it, isosteviol sodium is obtained which is a beyeranediterpene with therapeutic effects against diabetes mellitus, cardiovascular diseases, and cancer [151]. Yin et al. demonstrated a double effect of isosteviol sodium on cardiomyocytes [151]. It contrasted QTc prolongation related to ischemia/reperfusion injury, and it reduced Ikr and Ikatp channel inhibition during ischemia/reperfusion injury through the scavenging of ROS [151]. N-3 polyunsaturated fatty acids (PUFA) represent essential fatty acids which play an important role against cardiovascular diseases. N-3-PUFA, in particular docosahexaenoic acid (DHA) and eicosapentaenoic acid (EPA), are important activators of coronary smooth muscle cell BKCa channels contributing therefore to coronary vasodilation, in normal coronary arteries [152–154]. Tang et al. demonstrated that diabetic patients who have an impaired coronary ion channel function may have benefits, regarding cardiovascular complications, from N-3-PUFA assumption because they promote coronary BKCa channel activation and increased expression and they reduce Ca^2+^ concentration in coronary smooth muscle cells, increasing CBF [155]. Moreover, several antioxidant agents may contrast the effect of ROS and may preserve ion channel function. Several studies described the protective role of some NOX inhibitors against DM complications [156–163]. They may act as reducing ROS production, and among these are probucol [156], apocynin [157], plumbagin [158], and GLX351322 [159]. Gray et al. demonstrated the renal and atheroprotective effect of GKT 137831, a NOX1-4 inhibitor in insulin diabetes-deficient mouse [160]. It determines the reduction of atherosclerotic plaque diameter [160]. Nelson et al. demonstrated in a trial that the use of Protandim, an Nrf-2 activator, is associated with the induction of catalase and erythrocyte SOD activity in vivo and haem oxygenase 1 in vitro [161, 162]. Haem oxygenase 1 is an endogenous antioxidant which contrasts cardiomyocytes and endothelial apoptosis [161, 162]. Two different studies by Milman et al. and Blum et al. demonstrated that administration of vitamin E for 1.5 years may reduce cardiovascular events in patients with diabetes mellitus [163] (Table 2).

## 5. Conclusions

In conclusion, oxidative stress may represent a dangerous condition for organ and system function, and it is associated with several conditions such as diabetes mellitus, cardiovascular diseases, cancer, and neurological disorders. With this article, we aimed to investigate the physiological and pathophysiological role of oxidative stress in the connection between myocardial metabolism and CBF, with particular attention to patients with diabetes mellitus. There are several products of myocardial metabolism in the CBF regulation in relation with myocardial metabolic activity. However, the imbalance between oxidants and antioxidants, which defines the condition of oxidative stress, plays a crucial role in the alteration of CBF regulation in response to myocardial metabolism. In our previous studies [7–9], we already defined the importance of coronary ion channels as end effectors of CBF regulation mechanisms and the association between some SNPs of ion channel subunits and IHD. So, we investigated the impact of oxidative stress on ion channel function and the possibility to use them as therapeutic target in the treatment of IHD.

## Figures and Tables

**Table 1:** Coronary ion channels and their physiological role in the regulation of coronary vascular tone.

Ion channel	Membrane effect	Functions
Nav	Depolarization	Endothelial-dependent coronary vasodilation
Cl-	Depolarization	Endothelial-independent vasoconstriction
Cav	Depolarization	Coronary vasoconstriction; coronary autoregulation and control of coronary microvascular resistance
Kv	Hyperpolarization	Endothelial-dependent and -independent coronary vasodilation
KATP	Hyperpolarization	Coronary vasodilation and metabolic regulation of coronary blood flow
KCa	Hyperpolarization	Redox-sensitive channel, coronary dilatation in response to endothelial-derived hyperpolarizing factor (EDHF), lipoxygenase metabolites, and H_2_O_2_

**Table 2:** Main drugs and molecules which may have a role against IHD using ion channels as therapeutic target.

Drug	Biological effects	Functions
Sulphonylureas	Antagonist of the SUR subunit of pancreatic *β*-cell KATP	Promotion of *β*-cell depolarization and insulin secretion

Pinacidil and cromakalim	Kir6.2/SUR2A KATP and Kir6.2/SUR2B KATP channel openers	(i) Arteriole resistance reduction (ii) Arterial blood pressure reduction (iii) Vasodilatation

Nicorandil	Nitrate-like and proangiogenetic effect, calcium channel blocker, M2 macrophage polarization stimulator, M1 macrophage polarization inhibitor, and NF-*κ*B p65 subunit inhibitor	(i) Prevention of ventricular arrhythmias in patients who underwent coronary angioplasty after acute myocardial infarction (ii) Reduction of macrophage phagocytic activity, ROS and cytokine production, and improvement of mitochondrial membrane stability and Bcl-2/Bax ratio (iii) Promotion of endothelial reconstitution (iv) Improvement of the prognosis of patients with stable angina

Levosimendan	mBKCa-channel activator and calcium sensitizer, KATP activator	(i) Heart pump function improvement and reduction of the risk to develop arrhythmic events after myocardial ischemia (ii) Vasodilatation and increase in CBF

Isosteviol sodium	ROS scavenger	(i) Inhibition of QTc prolongation related to ischemia/reperfusion injury and reduction of I_kr_ and I_katp_ channel inhibition during ischemia/reperfusion injury

N-3-PUFA	Coronary BK_Ca_ channel activation, Ca^2+^ concentration in coronary smooth muscle cell reduction	(i) Vasodilation and increase in CBF

NOX inhibitor	ROS reduction	

Nrf-2 activator	Catalase and erythrocyte SOD activity induction in vivo	
Vitamin E	Antioxidant activity	
